# Optimizing the treatment of pain and anxiety in pediatric emergencies: the role of accreditation

**DOI:** 10.1186/s13584-019-0305-9

**Published:** 2019-04-08

**Authors:** Aaron Brody, Usha Sethuraman

**Affiliations:** Department of Emergency Medicine, Wayne State University School of Medicine, Detroit, MI USA

**Keywords:** Pain, Pediatric, Emergency, Pediatric emergency medicine, Accreditation

## Abstract

Pervasive disparities exist in the treatment of pain and anxiety in pediatric patients presenting to hospitals with emergency conditions. This finding has been demonstrated worldwide, and is especially exacerbated in general emergency departments, which treat both adults and children. Policies to promote appropriate analgesia in the context of pediatric emergency care have been developed by several professional societies and governmental agencies in the United States; however, progress has been uneven, and data regarding these questions is lacking.

In their excellent article, Capua and her co-authors address this precise problem through a unique methodology, by surveying nurse directors of both pediatric accredited and non-accredited emergency departments. Survey questions focused on availability of pharmacological and non-pharmacological modalities, and on the prevalence with which providers administered both oral and parenteral medications. The results demonstrated widespread availability of evidence based analgesic and anxiolytic treatment, ranging from medical clowns and specific holding positions, to use of intravenous opiates and conscious sedation. No significant differences were found associated with accreditation.

These results are surprising and seem to call into question the value of pediatric accreditation. However, an alternative hypothesis would be that accreditation has succeeded, and the results reflect a large spillover effect, in which providers trained in accredited institutions bring these advanced practices to their local departments. Regionalization has been promoted for emergency care of many acute conditions such as trauma, stroke, and myocardial infarction. These results suggest that for pediatric emergencies, at least in regard to analgesia, the answer likely lies in dissemination of knowledge, rather than super specialization. In other words, bring the expertise to the children, not the children to the experts. Further research in this area could focus on optimal ways to achieve such knowledge translation.

## Article

The care of children in the context of emergency medical services is a relatively new area of focus in emergency medicine research. A U.S. Institutes of Medicine report from 1993 [1] identified critical deficiencies in the care provided for children, and the preparedness of emergency departments (EDs) for true pediatric emergencies. Despite the proliferation of pediatric emergency departments (PEDs), and pediatric emergency medicine (PEM) training programs in the interval, a follow-up report in 2006 identified uneven progress in achieving these goals of quality pediatric emergency care [2]. Following the 2006 report, a joint society policy statement was developed by the American Academy of Pediatrics and the American College of Emergency Physicians, and endorsed by numerous other stakeholders, advocating for universal adoption of basic guidelines for optimal pediatric care [3]. The primary changes proposed include identification of physician and nursing leadership, maintaining appropriately sized equipment, and development of specific institutional protocols for pediatric emergency care. These principles were applied through the Emergency Medical Services for Children program, a U.S. federal grant program which funds PEM research, and provides guidance and support to PEDs. Although this program does not provide formal accreditation, it does provide training and performance assessment tools to regional health systems and individual hospitals [4].

In this context of optimizing pediatric emergency care, treatment of pain and anxiety has been identified as a priority [5]. Children are still less likely to receive proper analgesia than adults across populations and clinical conditions [6]. This disparity is exacerbated in general EDs, which treat both adults and children in the same location [7]. Some identified barriers to optimal pain management include lack of or inaccurate pain assessments [8–10], hesitation regarding the use of opioids in children among prescribers [11, 12], and failure to include non-pharmacologic strategies in the treatment of pain. However, interventions targeting improved documentation of pain scores did not translate into improved analgesic treatments [13, 14]. Racial disparities are associated with decreased utilization of analgesic medications [15–17]. This wide spectrum of factors leading to inadequate analgesia in the pediatric population leads to a conclusion that no single intervention is sufficient to solve this problem, and that only institutional culture change, with strong professional leadership can address the different barriers, and find effective solutions.

In their thoughtful and well written article [18], Dr. Capua and her colleagues evaluate the impact of PEM accreditation on the treatment of pain and anxiety among pediatric patients. The reality they describe, in which only half of the departments that treat children have formal accreditation, reflects the situation in the US, and worldwide, in which most children receive emergency care in non-specialized departments. The authors utilized a cross sectional survey of nurse managers of all the emergency departments EDs in Israel that accept pediatric patients (*n* = 21). Response rate to the survey was 95%. Half the respondents represented accredited departments (*n* = 10), and the remainder (*n* = 11) were from non-accredited institutions. Both types of departments provided a variety of pharmacological and non pharmacological interventions to alleviate pain and anxiety. Respondents for both types of institutions reported high utilization of certain non pharmacological modalities, such as medical clowns, sitting on parent’s lap during painful procedures, and music and decorations in procedural areas. No significant differences were noted in availability of oral and parenteral analgesics, but all of the pharmacological agents were used more frequently in accredited institutions.

These data would seem to suggest that pediatric accreditation only offers minor patient oriented benefits. However, the spill over effect, through dissemination of PEM trained *physicians* from the accredited facilities to non-accredited hospitals may have accounted for much of this parity. Furthermore, in a small country (population 8.8 million in 2018), with a closely knit medical community such as Israel, innovations from academic centers may diffuse quickly throughout the hospital ecosystem. Finally, the role of government regulation, such as the promotion of a nationally accredited protocol enabling nurses to administer acetaminophen and ibuprofen without a physician’s order, may have also encouraged non accredited centers to implement these interventions.

The difference noted in use of second line (i.e. opioid and parenteral analgesics) suggests PEM accreditation may improve patient care by one, or both of the following mechanisms: a) Clinicians in PEM accredited institutions are more aware of the subtle ways in which children may express undertreated pain, b) Physicians and staff in such institutions are better acquainted with the drugs and protocols required for sedation and opioid administration, and thus more liberal in their use.

The role of PEM accreditation and training in quality improvement and safety among trainees in improving the quality of pediatric emergency care has been previously described. Wong et al. [19] determined that while all curricular designs for trainees were effective in relaying knowledge and information regarding safety and quality improvement concepts, the curricula that demonstrated impact on clinical processes and patient care all had an experiential component. Mathias et al. [20]. described the incorporation of a peer review process into their PEM fellowship curriculum that included tracking of quality measures such as return visits, radiology errors and medication errors. Further, their PEM fellows actively participated (with an assigned faculty member) in the development of evidence based protocols for management of various disease processes such as bronchiolitis, and quality improvement projects for pain management of long bone fractures and sickle cell pain crisis thus helping streamline and standardize care in an otherwise chaotic environment. Such involvement of fellows in quality improvement measures including that of pain control provides them with the knowledge and tools necessary to incorporate evidence based care into their independent practice through experiential training.

The role of institutional accreditation as a means to improve patient care has not been described previously in the context of pediatric pain management. This study adds a significant contribution to existing literature regarding the positive role of accreditation in treating critical conditions such as myocardial infarction, cardiology, stroke, and trauma [21, 22]. Among the many factors contributing to these advances, designation of professional leadership, implementation of protocol based therapy, availability of specialized equipment, and institutional commitment to improving measurable outcomes are the most salient. The outcome of such institutional accreditation is often regionalization of healthcare. However, the present study suggests that non accredited institutions can also optimize care in specific scenarios, thus sparing patients and families the time, expense, and delay in treatment associated with transport to specialized centers. This message is of great import in areas where community hospitals with general EDs that treat children far outnumber accredited PEDs. Thus, in contrast to the diseases and conditions mentioned above, the goal for PEM should not be increased specialization. Dissemination of best practices from referral centers to community EDs through physician training programs and other forms of outreach will likely provide the best and most accessible care for sick and injured children.

## Conclusions

In summary, the authors describe an unexpected finding, that PEM accreditation does not have a significant impact on patient level analgesic strategies. However, these data also suggest that dissemination of these advanced practices has already happened, and this is an important lesson for other health care system stakeholders seeking to optimize PED care. Future research should address the ideal mechanisms to disseminate knowledge within PEM.

## Abbreviations

ED: Emergency Department
PED: Pediatric Emergency Department
PEM: Pediatric Emergency Medicine
US: United States
